# Clinical and radiological outcomes of directed treatment transitions from Gilenya^®^ to generic fingolimod

**DOI:** 10.1177/13524585251401404

**Published:** 2026-01-03

**Authors:** Mahi A Patel, Tom G Punnen, Kevin S Shan, Morgan C McCreary, Crystal M Wright, Shanan B Munoz, Paula Hardeman, Katy W Burgess, Benjamin M Greenberg, Lindsay A Horton, Peter V Sguigna, Lauren M Tardo, Olaf Stüve, Darin T Okuda

**Affiliations:** College of Osteopathic Medicine, Kansas City University, Kansas City, MO, USA; School of Medicine, The University of Texas Southwestern Medical Center, Dallas, TX, USA; School of Medicine, The University of Texas Southwestern Medical Center, Dallas, TX, USA; Department of Neurology, The University of Texas Southwestern Medical Center, Dallas, TX, USA; Peter O’Donnell Jr. Brain Institute, The University of Texas Southwestern Medical Center, Dallas, TX, USA; Department of Neurology, The University of Texas Southwestern Medical Center, Dallas, TX, USA; Peter O’Donnell Jr. Brain Institute, The University of Texas Southwestern Medical Center, Dallas, TX, USA; Department of Neurology, The University of Texas Southwestern Medical Center, Dallas, TX, USA; Peter O’Donnell Jr. Brain Institute, The University of Texas Southwestern Medical Center, Dallas, TX, USA; Department of Neurology, The University of Texas Southwestern Medical Center, Dallas, TX, USA; Peter O’Donnell Jr. Brain Institute, The University of Texas Southwestern Medical Center, Dallas, TX, USA; Department of Neurology, The University of Texas Southwestern Medical Center, Dallas, TX, USA; Peter O’Donnell Jr. Brain Institute, The University of Texas Southwestern Medical Center, Dallas, TX, USA; Department of Neurology, The University of Texas Southwestern Medical Center, Dallas, TX, USA; Peter O’Donnell Jr. Brain Institute, The University of Texas Southwestern Medical Center, Dallas, TX, USA; Department of Neurology, The University of Texas Southwestern Medical Center, Dallas, TX, USA; Peter O’Donnell Jr. Brain Institute, The University of Texas Southwestern Medical Center, Dallas, TX, USA; Department of Neurology, The University of Texas Southwestern Medical Center, Dallas, TX, USA; Peter O’Donnell Jr. Brain Institute, The University of Texas Southwestern Medical Center, Dallas, TX, USA; Department of Neurology, The University of Texas Southwestern Medical Center, Dallas, TX, USA; Peter O’Donnell Jr. Brain Institute, The University of Texas Southwestern Medical Center, Dallas, TX, USA; Department of Neurology, The University of Texas Southwestern Medical Center, Dallas, TX, USA; Peter O’Donnell Jr. Brain Institute, The University of Texas Southwestern Medical Center, Dallas, TX, USA; Neurology Section, Dallas Veterans Affairs Medical Center, Dallas, TX, USA; Department of Neurology, The University of Texas Southwestern Medical Center, Dallas, TX, USA; Peter O’Donnell Jr. Brain Institute, The University of Texas Southwestern Medical Center, Dallas, TX, USA

**Keywords:** Multiple sclerosis, fingolimod, disease-modifying therapies, MRI, absolute lymphocyte count

## Abstract

**Background:**

Gilenya^®^ (fingolimod), a sphingosine-1-receptor agonist, is an effective treatment for multiple sclerosis (MS). However, increased relapse activity has been observed after transitioning to generic fingolimod despite prior prolonged disease stability.

**Objective:**

To quantify the clinical and radiological impact of transitioning from Gilenya^®^ to generic fingolimod in people with MS (PwMS).

**Methods:**

Retrospective data were evaluated from a single tertiary MS care center. Time to magnetic resonance imaging (MRI) activity and clinical relapse was assessed during Gilenya^®^ and generic fingolimod treatment. Differences in absolute lymphocyte count (ALC) and side effects were also measured by treatment group.

**Results:**

The cohort included 88 PwMS (71 female; 76 White, mean age when starting Gilenya^®^ was 39.6 years (standard deviation (SD) = 10.6 years), and mean age when starting generic fingolimod was 46.9 years (SD = 11.2 years)). A shorter time to MRI activity (*p* = 0.0026) and time to relapse (*p* = 0.0027) was observed during generic fingolimod treatment. The ALC increased by 8.81% after generic fingolimod treatment, relative to Gilenya^®^ (95% CI = (2.00%, 16.08%), *p* = 0.01), with an intersubject variability of 1.97%. A 2.45-fold increase in side effects was observed with generic fingolimod relative to Gilenya^®^ (95% CI = (1.38, 4.36), *p* = 0.002).

**Conclusion:**

Measures of disease stability appear less optimal with generic fingolimod based on serological, clinical, and radiological measures.

## Introduction

Multiple sclerosis (MS) is a chronic, demyelinating condition of the central nervous system (CNS) involving autoinflammatory attacks mediated by lymphocytes and monocytes that cross the blood–brain barrier.^1^ The clinical manifestation of this condition encompasses a wide range of experiences, including visual impairment, sensory disturbances, motor weakness, cognitive impairment, or no symptoms.^2^ Disease-modifying therapy (DMT) exposure prior to the development of a first clinical symptom,^3,4^ in addition to continued treatment in those with established and stable disease, may improve longer-term clinical outcomes.^5^

Currently, there are three oral DMTs for MS available in generic form: fingolimod, dimethyl fumarate, and teriflunomide. Gilenya^®^ (fingolimod) is a sphingosine-1-phosphate (S1P) modulator that was approved by the United States Food and Drug Administration (FDA) on 22 September 2010, for use in individuals with MS. This medication works to reduce disease activity by restricting lymphocyte egress from the lymph nodes^6^ and was found to be effective in two pivotal trials.^6,7^ Fingolimod 0.5 mg is the only FDA approved dosage in adults after comparing the efficacy and safety of 0.25 mg and 1.25 mg capsules.^8–10^ Given the mechanism of action of fingolimod, a consistent dosage regimen is required to provide optimal effect and discontinuation of treatment has been linked to disease rebound.^11^

Generic fingolimod was approved by the US FDA on 5 December 2019, for people with relapsing forms of MS. In 2023, third-party administrators directed people with MS (PwMS) to transition from Gilenya^®^ to the generic alternative, despite having no history of disease advancement, indicators of adverse reactions, or treatment intolerance. Consumers also faced higher treatment costs with the generic version due to the absence of manufacturer support. Major recalls of generic therapies by government agencies due to high variability in capsule content and failure to comply with Current Good Manufacturing Practices (cGMP) have raised questions regarding the quality, reliability, and efficacy of these therapies.^12–15^ Recent data suggested that inconsistencies in the potency of generic fingolimod may lead to disease worsening and increased patient harm.^16^ Despite these findings, larger case series studying patient outcomes on generic fingolimod have not been reported.

In this study, we aimed to quantify clinical, radiological, and biological outcomes of directed treatment transitions by third-party administrators from Gilenya^®^ to generic fingolimod in PwMS. We hypothesized that elevated absolute lymphocyte counts (ALCs), higher relapse events, reduced infections, and differences in treatment tolerance may be observed after transitioning to generic fingolimod compared to prior experiences on Gilenya^®^ based on concern for lower dosages due to variable manufacturing. If present, a transformation in the routine clinical surveillance for those with MS may be required, especially if exposure to generic alternatives is associated with greater disease advancement.

## Methods

### Research participants

The study group was obtained from the University of Texas Southwestern Medical Center (UTSW) at Dallas, Multiple Sclerosis and Neuroimmunology Clinic. Participants were people being followed for care along with new consultations. PwMS who were treated with Gilenya^®^ or generic fingolimod were identified after review of electronic medical records for individuals with 340.0 ICD-9 or G35.0 ICD-10 codes from 2023 to 2024.

**Figure 1.**
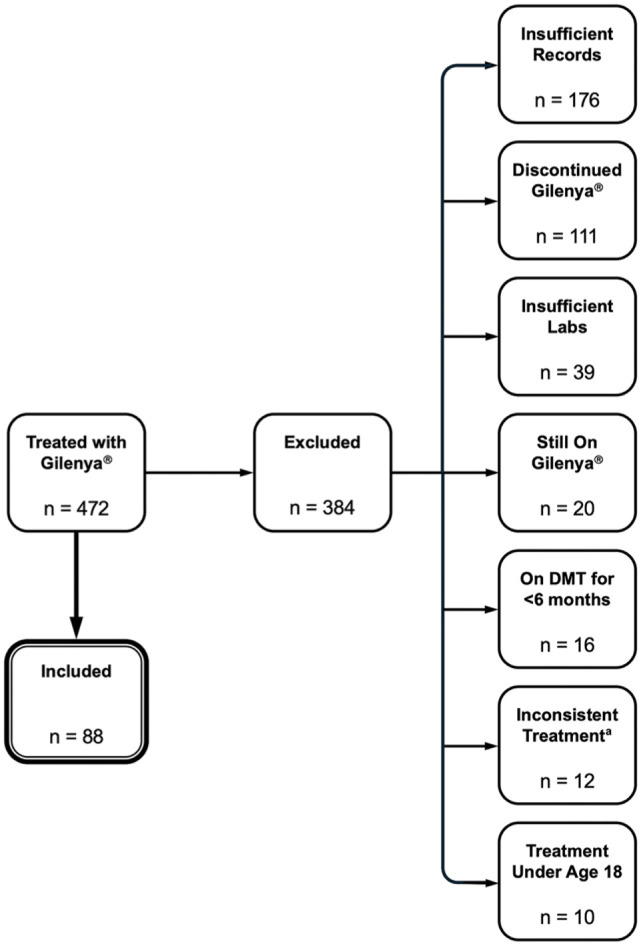
Flowchart diagram illustrating the breakdown of included and excluded subjects. ^a^Gaps due to pregnancy (*n* = 3), insurance accessibility (*n* = 2), treatment non-compliance (*n* = 2), and inconsistent dosage (*n* = 5).

The inclusion criteria involved (1) males and females over the age of 18 with (2) an established diagnosis of MS fulfilling 2017 McDonald Criteria^17^ following a comprehensive medical evaluation by fellowship-trained specialists in neuroimmunology who (3) were treated with Gilenya^®^ for at least 1 year prior to transitioning to generic fingolimod with a treatment exposure period of at least 6 months, with (4) absolute lymphocyte data available for analysis while on Gilenya^®^ and at 8 weeks after transitioning to generic fingolimod. Subjects were excluded if (1) they had insufficient laboratory and/or medical record data, (2) began treatment on Gilenya^®^ prior to the age of 18, (3) exhibited non-compliance with treatment, or (4) experienced interruptions in treatment while on generic fingolimod. The distribution of included and excluded subjects is shown in Figure 1.

The protocol was approved by the UTSW Institutional Review Board and has, therefore, been performed in accordance with the ethical standards laid down in the 1964 Declaration of Helsinki and later amendments. Consent was acquired from all included individuals.

### Data collection

For each subject, demographic data, and medical history including reported infections, described side effects, clinical relapse activity, magnetic resonance imaging (MRI) activity (i.e. gadolinium-enhancing, new and/or newly enlarging T2-weighted hyperintense lesions), DMT treatment history, and laboratory results, including complete blood count values containing ALCs, were collected for up to 2 years prior to their transition to generic fingolimod until November 2024. Details regarding adverse reactions were captured from all clinical encounters, including data from within and outside of clinical visits, documented within the electronic medical record.

### Statistical analysis

A zero-inflated Poisson mixed model with an offset term corresponding to the duration on the respective DMT was estimated with a covariate corresponding to treatment to assess if there was a difference in the number of side effects on Gilenya^®^ versus on generic fingolimod for a given treatment duration. A zero-inflated Poisson mixed-effects model was also estimated with a covariate corresponding to treatment and an offset term corresponding to the duration on the respective DMT to test for differences in the number of infections per year between Gilenya^®^ and generic fingolimod for a given treatment duration.

To measure differences in ALC values when subjects were on Gilenya^®^ versus generic fingolimod, a linear mixed-effects model was estimated with the dependent variable corresponding to the log base 10 lymphocyte count and covariates defined as time from the respective DMT initiation, treatment (Gilenya^®^ vs. generic fingolimod), and the interaction of time from DMT initiation and treatment.

To estimate the time to new MRI activity/relapse and testing for differences in the time to new MRI activity/relapse by treatment, Kaplan–Meier curves were estimated for the time to new MRI activity/relapse and a log-rank test was used to assess for differences between treatments while accounting for right censoring. Right censoring corresponds to a participant terminating treatment before experiencing an event (e.g. MRI activity or relapse) or reaching the final date of follow-up without experiencing an event.

All analyses were performed in R (version 4.4.3). All plots were generated using the ggplot2 package. Due to the exploratory nature of the analyses, *p*-values were not adjusted to account for multiple testing. A *p*-value < 0.05 was considered significant.

## Results

The cohort included 88 PwMS (71 female; 76 White), having initial exposure to Gilenya^®^ with subsequent directed transition to generic fingolimod. The mean age when starting Gilenya^®^ was 39.6 years (SD = 10.6 years) and 46.9 years (SD = 11.2 years) at the start of generic fingolimod. The mean disease duration from time of first symptom was 17.2 years (SD = 7.9 years), and the mean time from diagnosis was 15.3 years (SD = 6.9 years). For all individuals, the mean total duration of treatment was 7.0 years (SD = 3.1 years) for Gilenya^®^ and 1.6 years (SD = 0.5 years) for generic fingolimod. A majority of the cohort were previously treated with a DMT prior to transitioning to Gilenya^®^ (69.3%) and were insured under Blue Cross Blue Shield of Texas at the time of directed transition to generic fingolimod (55.7%). Table 1 provides further demographic details of the study cohort.

**Table 1.** Baseline demographic data.

n	Overall
	88
Gilenya^®^ age	
Mean (SD)	39.6 (10.6)
Generic fingolimod age	
Mean (SD)	46.9 (11.2)
Ethnicity	
Hispanic	5 (5.7%)
Non-Hispanic	83 (94.3%)
Race	
African American	11 (12.5%)
Asian	1 (1.1%)
White	76 (86.4%)
Insurance provider	
Aetna	6 (6.8%)
Blue Cross Blue Shield of Texas	49 (55.7%)
Cigna	5 (5.7%)
Generic exchange	1 (1.1%)
Humana	2 (2.3%)
Medicare/Medicaid	9 (10.2%)
None	1 (1.1%)
Other	1 (1.1%)
United healthcare	12 (13.6%)
Unknown	2 (2.3%)
Previous DMT	
Dimethyl fumarate (Tecfidera^®^)	11 (12.5%)
Diroximel fumarate (Vumerity^®^)	1 (1.1%)
Glatiramer acetate (Copaxone^®^)	17 (19.3%)
Interferon beta-1a (Avonex^®^)	10 (11.4%)
Interferon beta-1a (Rebif^®^)	10 (11.4%)
Interferon beta-1b (Betaseron^®^)	2 (2.3%)
Natalizumab (Tysabri^®^)	5 (5.7%)
None	27 (30.7%)
Non-DMT therapy	1 (1.1%)
Peginterferon beta-1a (Plegridy^®^)	1 (1.1%)
Rituximab (Rituxan^®^)	1 (1.1%)
Teriflunomide (Aubagio^®^)	2 (2.3%)

There was a 2.45-fold increase in the rate of side effects per year while on generic fingolimod relative to Gilenya^®^ (95% confidence interval (CI) =(1.38, 4.36), *p* = 0.002). Observed side effects included headache, weight gain, and sensory changes/myalgia. Individuals exposed to Gilenya^®^ were observed with a higher infection rate, complaints of bradycardia, and gastrointestinal experiences when compared to generic fingolimod. A comprehensive list of all adverse reactions is provided in Table 2.

**Table 2.** Adverse reactions reported while on Gilenya^®^ versus on generic fingolimod.

n	Generic	Gilenya^®^	Overall
	88	88	176
Average number of side effects (SD)	0.216 (0.535)	0.386 (0.633)	0.301 (0.591)
ALC ⩽ 200	1 (1.1%)	3 (3.4%)	4 (2.3%)
Poor appetite	1 (1.1%)	0 (0%)	1 (0.6%)
Cold symptoms	1 (1.1%)	4 (4.5%)	5 (2.8%)
Weight gain	2 (2.3%)	1 (1.1%)	3 (1.7%)
Increased infections	0 (0%)	3 (3.4%)	3 (1.7%)
Bradycardia	0 (0%)	4 (4.5%)	4 (2.3%)
Headache	3 (3.4%)	5 (5.7%)	8 (4.5%)
Fatigue	0 (0%)	3 (3.4%)	3 (1.7%)
Stomach/diarrhea	0 (0%)	3 (3.4%)	3 (1.7%)
Hair loss	0 (0%)	1 (1.1%)	1 (0.6%)
Vision	1 (1.1%)	2 (2.3%)	3 (1.7%)
Dermatological effects	0 (0%)	2 (2.3%)	2 (1.1%)
Elevated ALTs	0 (0%)	1 (1.1%)	1 (0.6%)
Syncope	0 (0%)	1 (1.1%)	1 (0.6%)
Dizziness	0 (0%)	1 (1.1%)	1 (0.6%)
BP became normal	1 (1.1%)	0 (0%)	1 (0.6%)
Macular edema	1 (1.1%)	0 (0%)	1 (0.6%)
Decreased infections	1 (1.1%)	0 (0%)	1 (0.6%)
Twitching	1 (1.1%)	0 (0%)	1 (0.6%)
Sensory symptoms/myalgia	3 (3.4%)	1 (1.1%)	4 (2.3%)
Trouble focusing	1 (1.1%)	0 (0%)	1 (0.6%)
Foot drop/numbness	1 (1.1%)	0 (0%)	1 (0.6%)
Increased BP	0 (0%)	2 (2.3%)	2 (1.1%)

The ALC increased by 8.81% while on generic fingolimod relative to Gilenya^®^ (95% CI = (2.00%, 16.08%), *p* = 0.01) with intersubject variability estimated to be 1.97%. In addition, significant changes in ALC over time were not observed while on Gilenya^®^ (*p* = 0.18) or on generic fingolimod (*p* = 0.45).

The majority of relapses were identified following directed transition to generic fingolimod (*n* = 10) in comparison to the time while individuals were treated with Gilenya^®^ (*n* = 2). This was still observed despite the longer average treatment duration for the two individuals on Gilenya^®^ prior to relapse (2.0 years (SD = 2.8 years)), compared to the average treatment duration for the 10 individuals on generic fingolimod prior to relapse (0.8 years (SD = 0.4 years)). In addition, subjects who experienced relapses on generic fingolimod were stable on treatment with Gilenya^®^ for an average of 5.8 years (SD = 3.5 years) before transitioning to generic fingolimod. Despite the shorter treatment duration, the time to new MRI activity (*p* = 0.0026) and time to relapse (*p* = 0.0027) were significantly shorter during treatment with generic fingolimod compared to those who experienced disease activity on Gilenya^®^. However, given the limited number of participants who experienced MRI activity while on either treatment, determining an estimate for the median survival time was not possible.

Individuals with disease progression after transitioning to generic fingolimod either experienced symptoms coupled with corresponding new changes on MRI (*n* = 5) or the development of new MRI activity consistent with inflammatory demyelination (*n* = 5). For the individuals who experienced a relapse on Gilenya^®^ (*n* = 2), new MRI changes were observed in the absence of clinical symptoms. Table 3 provides the statistics for relapse occurrence and MRI activity data. Regardless of their treatment group, ALCs were elevated in seven subjects (Gilenya^®^ = 1, generic fingolimod = 6) at the time of relapse compared to during disease stability.

**Table 3.** Summary statistics for MRI activity and relapse occurrence.

n	Generic	Gilenya^®^	Overall
	88	88	176
MRI activity, *n* (%)	10 (11.4%)	2 (2.3%)	12 (6.8%)
Average years to MRI activity or censoring (SD)	1.43 (0.54)	6.66 (3.44)	4.05 (3.59)
MRI activity location, *n* (%)			
Brain	6 (6.8%)	1 (1.1%)	7 (4.0%)
Cervical spine	3 (3.4%)	1 (1.1%)	4 (2.3%)
Brain & cervical spine	1 (1.1%)	0 (0%)	1 (0.6%)
MRI classification, *n* (%)			
New T2 lesions	4 (4.5%)	2 (2.3%)	6 (3.4%)
Enlarging T2 lesions	2 (2.3%)	0 (0%)	2 (1.1%)
Enhancement	1 (1.1%)	0 (0%)	1 (0.6%)
New and enhancing lesions	3 (3.4%)	0 (0%)	3 (1.7%)
Relapse, *n* (%)	10 (11.4%)	2 (2.3%)	12 (6.8%)
Average years to relapse or censoring (SD)	1.42 (0.55)	6.66 (3.44)	4.04 (3.59)
Relapse type, *n* (%)			
MRI disease activity (no clinical symptoms)	5 (5.7%)	2 (2.3%)	7 (4.0%)
Clinical relapse (symptoms only)	2 (2.3%)	0 (0%)	2 (1.1%)
Clinical and MRI	3 (3.4%)	0 (0%)	3 (1.7%)
Clinical relapse type, *n* (%)			
Motor	1 (1.1%)	0 (0%)	1 (0.6%)
Sensory	4 (4.5%)	0 (0%)	4 (2.3%)

## Discussion

In this study, we identified that subjects had over a twofold increase in side effects and almost 9% increase in ALC following a directed transition to generic fingolimod compared to their experience on Gilenya^®^. A significant difference in time to relapse was also identified, with a shorter average time to relapse on generic fingolimod observed when compared to Gilenya^®^. Our results indicate that variations in the clinical effects of generic fingolimod may be present, supported by the higher relapse rate, increased side effects, and elevations in ALCs compared to their experience on Gilenya^®^.

Phosphorylated fingolimod (FTY720) acts as an agonist for sphingosine receptors S1P_1_, S1P_3_, S1P_4_, and S1P_5_.^18^ This reaction prevents the egress of lymphocytes out of the lymph nodes, subsequently reducing the risk of autoinflammatory reactions as fewer autoreactive lymphocytes are able to enter the CNS.^6,18^ Due to the mechanism of the drug, people who discontinue fingolimod face the risk of disease rebound.^11^ Rates of severe relapse in people range between 10% and 25%, with onset beginning as soon as 3 weeks post-treatment. On average, rebound activity may occur within 2–4 months after treatment cessation.^19^ In this study, 11% of subjects experienced relapse between 2 and 16 months after transitioning to generic fingolimod. Our observed results may be related to these phenomena, given the timing of the documented clinical and radiological events. As the half-life of fingolimod is 6–9 days, a single month of treatment with generic fingolimod that may be suboptimal in effect may leave people vulnerable to new disease activity.^16,19^

The assessment of ALC values indicates the potential effect of an S1P-targeted therapy. Over half of the subjects who relapsed on generic fingolimod experienced an elevation in ALCs before the event. Although higher ALCs on Gilenya^®^ were not associated with decreased treatment efficacy or increased relapse rates in prior published studies, a relationship between higher ALC and increased risk of disease activity was identified in a study with dimethyl fumarate, suggesting a connection between treatment efficacy and ALC.^20–22^ In addition, these trials were conducted to study the safety and efficacy of Gilenya^®^, prior to the availability of the generic version. Biological variation may induce varying degrees of reduction in ALC; however, intrasubject variability, as observed in this study, gives cause for appropriate concern, especially following the start of treatment with generic fingolimod. Subsequent to the commercial distribution of generic fingolimod, increased disease progression has also been observed during treatment with the generic in comparison to Gilenya^®^, where 12 subjects from a cohort of 27 had increases in Expanded Disability Status Scale scores within 1 year of treatment with generic fingolimod compared to four subjects within 1 year on Gilenya^®^. In addition, this study revealed that 17 of the 27 subjects had to discontinue treatment with generic fingolimod and switched back to Gilenya^®^ after experiencing new/worsening side effects (*n* = 13) and relapse activity (*n* = 4).^23^ This investigation provides further support for these findings, as both increased side effects and new disease activity were observed in patients after transitioning to generic fingolimod in this study.

The process of creating generic drugs involves reverse-engineering the brand-name product. Brand companies often patent technical steps of their manufacturing process, such as their time-release mechanism, causing generic manufacturers to use differing excipients, or fillers, and dyes.^24^ Differences in excipients between manufacturers may lead to inconsistent dissolution rates and levels of degradation.^25^ In addition, certain bulking agents may be less tolerated by certain people and cause additional side effects, especially in the majority of PwMS with concomitant gastrointestinal syndromes.^26^ For example, pregelatinized starch and maltodextrin may be extracted from wheat and can cause adverse reactions in people with intolerance to gluten.^27^ Propylene glycol has also been associated with adverse reactions.^28,29^ These variabilities may explain the more than twofold increase in side effects reported by subjects after switching to generic fingolimod. Despite the increase in the average number of reported side effects per year on generic fingolimod compared to Gilenya^®^, 30% of side effects described while on generic fingolimod were not described in patients on Gilenya^®^. Although these results may be influenced by the longer treatment durations on Gilenya^®^, they reveal potential differences in the mechanistic effect of variable generic products in comparison to the brand name. A comprehensive list of inactive ingredients included in fingolimod capsules across various manufacturers is provided in Table 4.

**Table 4.** Inactive ingredients in capsules from different manufacturers of fingolimod.

Drug	Manufacturer	Inactive ingredients
Gilenya^®^ (0.5 mg)	Novartis AG	Gelatin, Magnesium Stearate, Mannitol, Titanium Dioxide, and Yellow Iron Oxide.^a^
Generic fingolimod (0.5 mg)	Accord Healthcare Inc.	Gelatin, Magnesium Stearate, Pregelatinized Starch (Maize), Titanium Dioxide, Yellow Iron Oxide. Imprinting Ink: Black Iron Oxide, Potassium Hydroxide, Shellac, Yellow Iron Oxide.^b^
	Apotex Corp.	Fumaric Acid, Gelatin, Stearic Acid, Pregelatinized Starch, Titanium Dioxide. Imprinting Ink: Black Iron Oxide, Potassium Hydroxide, Propylene Glycol, Shellac.^c^
	AvKARE Inc.	Gelatin, Pregelatinized Starch (Corn), Titanium Dioxide, Sodium Lauryl Sulfate. Imprinting Ink: Black Iron Oxide, Potassium Hydroxide, Propylene Glycol, Shellac, Strong Ammonia Solution.^d^
	Aurobindo Pharma Ltd.	Black Iron Oxide, Gelatin, Maltodextrin, Sodium Stearyl fumarate, Talc, Titanium Dioxide, Yellow Iron Oxide. Imprinting Ink: Black Iron Oxide, Potassium Hydroxide, Propylene Glycol, Shellac, Strong Ammonia Solution.^e^
	Camber Pharmaceuticals Inc.	Black Iron Oxide, FD&C Blue #2 Aluminum Lake, Gelatin, Magnesium Stearate, Potassium Hydroxide, Powdered Cellulose, Propylene Glycol, Shellac, Sodium Lauryl Sulfate, Titanium Dioxide, Yellow Iron Oxide.^f^
	Dr. Reddy’s Laboratories Inc.	Betacyclodextrin, Black Iron Oxide, Dehydrated Alcohol, Gelatin, Magnesium Stearate, Potassium Hydroxide, Propylene Glycol, Shellac, Sodium Lauryl Sulfate, Strong Ammonia Solution, Titanium Dioxide, and Yellow Iron Oxide.^g^
	Glenmark Pharmaceuticals Inc., USA	Gelatin, Magnesium Stearate, Pregelatinized Starch, Titanium Dioxide, Yellow Iron Oxide. Imprinting Ink: Black Iron Oxide, Potassium Hydroxide, and Shellac.^h^
	Mylan Pharmaceuticals Inc.	Colloidal Silicon Dioxide, Dibasic Calcium Phosphate Dihydrate, Glycine, Magnesium Stearate, Phosphorus, Red Iron Oxide, Sodium Lauryl Sulfate, Titanium Dioxide, and Yellow Iron Oxide. Imprinting Ink: Black Iron Oxide, Potassium Hydroxide, Propylene Glycol, Shellac, and Strong Ammonia Solution.^i^
	Quallent Pharmaceuticals Health LLC	Colloidal Silicon Dioxide, Gelatin, Magnesium Stearate, Microcrystalline Cellulose, Titanium Dioxide, Yellow Iron Oxide. Imprinting Ink: Black Iron Oxide, Potassium hydroxide, Shellac.^j^
	Solco Healthcare US LLC	Black Iron Oxide, Gelatin, Magnesium Stearate, Potassium Hydroxide, Propylene Glycol, Silicified Microcrystalline Cellulose, Shellac and Titanium Dioxide.^k^
	Sun Pharmaceutical Industries Ltd.	Colloidal Silicon Dioxide, Crospovidone, Gelatin, Magnesium Stearate, Polacrilin Potassium, Sodium Lauryl Sulfate, Titanium Dioxide, Water, and Yellow Iron Oxide. Imprinting Ink: Black Iron Oxide, Butyl Alcohol, Potassium Hydroxide, Propylene Glycol, Shellac, and Strong Ammonia Solution.^l^
	Rising Pharma Holdings Inc.	Gelatin, Magnesium Stearate, Pregelatinized Starch, Titanium Dioxide. Imprinting Ink: Black Iron Oxide, Potassium Hydroxide, Shellac.^m^
	Teva Pharmaceuticals Inc.	Gelatin, Pregelatinized Starch (Corn), Sodium Lauryl Sulfate, Titanium Dioxide, Yellow Iron Oxide. Imprinting Ink: Black Iron Oxide, Potassium Hydroxide, Propylene Glycol, Shellac, and Strong Ammonia Solution.^n^
	XLCare Pharmaceuticals Inc.	Black Iron Oxide, FD&C Blue # 2 Aluminum Lake, Gelatin, Magnesium Stearate, Potassium Hydroxide, Powdered Cellulose, Propylene Glycol, Shellac, Sodium Lauryl Sulfate, Titanium Dioxide, and Yellow Iron Oxide.^o^
	Zydus Pharmaceuticals USA Inc.	Gelatin, Pregelatinized Starch (botanical source: maize), Sodium Lauryl Sulfate, Sodium Stearyl Fumarate, Titanium Dioxide, Yellow Iron Oxide, Water. Imprinting ink: Black Iron Oxide, Potassium Hydroxide, Propylene Glycol, Purified Water, Shellac.^p^

a Gilenya^®^ [package insert]. East Hanover, NJ, USA: Novartis Pharmaceuticals Corporation; 2025.

b Fingolimod [package insert]. Ahmedabad, GJ, India: Accord Healthcare Inc.; 2024.

c Fingolimod [package insert]. TG, India: Apotex Corp.; 2024.

d Fingolimod [package insert]. Pulaski, TN, USA: AvKARE Inc.; 2024.

e Fingolimod [package insert]. Hyderabad, TG, India: Aurobindo Pharma Ltd.; 2024.

f Fingolimod [package insert]. Jeedimetla, TG, India, or Hauppauge, NY, USA: Camber Pharmaceuticals Inc.; 2024.

g Fingolimod [package insert]. India: Dr. Reddy’s Laboratories Inc.; 2024.

h Fingolimod [package insert]. Pithampur, MP, India: Glenmark Pharmaceuticals Inc., USA; 2024.

i Fingolimod [package insert]. Hyderabad, TG, India: Mylan Pharmaceuticals Inc.; 2024.

j Fingolimod [package insert]. Bengaluru, KA, India: Quallent Pharmaceuticals Health LLC; 2024.

k Fingolimod [package insert]. Linhai, ZJ, China: Solco Healthcare US LLC; 2024.

l Fingolimod [package insert]. Halol, GJ, India: Sun Pharmaceutical Industries Ltd.; 2024.

m Fingolimod [package insert]. Dongguan, GD, China: Rising Pharma Holdings Inc.; 2024.

n Fingolimod [package insert]. Kfar Saba, Sharon Region, Israel: Teva Pharmaceuticals Inc.; 2024.

o Fingolimod [package insert]. Hauppauge, NY, USA: XLCare Pharmaceuticals Inc.; 2024.

p Fingolimod [package insert]. Ahmedabad, GJ, India: Zydus Pharmaceuticals USA Inc.; 2024.

In the United States, 90% of filled prescriptions comprise generic drugs.^30^ To distribute their products in the United States, pharmaceutical manufacturers are instructed to meet FDA benchmarks requiring generic drugs to contain within 90%–110% of stated dosage of the active pharmaceutical ingredient, and an 80%–125% bioequivalence range. Observed deficiencies in generic drugs may result from various types of non-compliance with FDA cGMP.^31^ Of the 13 pharmaceutical companies approved to manufacture generic fingolimod, 6 are located abroad. Foreign manufacturing plants are required to adhere to the same cGMP guidelines applied to domestic plants. However, unlike facilities in the United States, which can be subject to inspections at any time without notice, foreign inspections must be scheduled many months in advance.^32^ In the United States, the risk of surprise inspections may provide facilities increased incentive to remain in strict accordance with cGMP guidelines, but it is difficult to carry this sentiment abroad due to lack of imminent surveillance, which may reduce adherence practices.

This work should be reviewed in the context of limitations. This study was performed through retrospective review of medical records; therefore, intervals between clinical follow-up, lab testing, and the timing of MRI studies varied. Both patient- and physician-reporter bias may have influenced the reported findings. The risk of potential informative censoring related to immortal time bias may also have been a factor, as those on Gilenya^®^ were followed over four times longer than individuals on generic fingolimod. If disease activity or adverse reactions occurred, a transition to another DMT would have likely taken place. In addition, the source of generic fingolimod received monthly by people remains unknown. Our direct clinical experience reveals that individuals are exposed to numerous generic manufacturers throughout the year. Considering the inconsistent sourcing of their medications and the established variability in quality of generic medications, the negative effects of a substandard batch of therapies may not manifest immediately.^14,16^ Without direct examination of the treatment capsules, the observed disease activity cannot be directly attributed to the efficacy of generic fingolimod. However, the results of this study provide serological and clinical context to support the observed clinical relapse and MRI activity findings. Due to the nature of prescription authorizations by third-party administrators, people were transitioned to generic fingolimod at varying time points and the duration of follow-up was not as robust for those taking generic fingolimod. Therefore, clinical data while on generic treatment were not as extensive as while on Gilenya^®^. Data were only collected from the last 2 years of treatment on Gilenya^®^ before switching to generic fingolimod to balance the difference in treatment timelines.

The majority of subjects in this cohort have remained clinically and radiologically stable on generic fingolimod. However, several individuals with a history of disease stability on Gilenya^®^ demonstrated worsening upon transitioning to generic treatment. Our findings suggest the importance of clinical surveillance when PwMS are transitioned from Gilenya^®^ to generic fingolimod. They also argue against the presumption of uniform treatment quality between the Gilenya^®^ and generic fingolimod. The use of generic DMTs is commonly required by third-party administrators when individuals are newly diagnosed. As exposure to such treatments is anticipated to continue, clinical documentation of the manufacturer, along with vigilant monitoring through follow-up visits, blood studies, and MRI surveillance, should be considered to evaluate treatment-related events.
